# Redox-Immune Axis and Ozone Pollution: From Oxidative Stress to Thymic Involution and Neurodegeneration

**DOI:** 10.3390/medsci13040293

**Published:** 2025-11-29

**Authors:** Marlen Valdés-Fuentes, Erika Rodríguez-Martínez, Selva Rivas-Arancibia

**Affiliations:** Departamento de Fisiología, Facultad de Medicina, Universidad Nacional Autónoma de México, Coyoacán, Mexico City 04510, Mexico; marlen_valdes@ciencias.unam.mx (M.V.-F.); arodriguez@facmed.unam.mx (E.R.-M.)

**Keywords:** oxidative stress, thymic senescence, thymic inflammaging, neurodegenerative disease

## Abstract

Chronic exposure to low concentrations of ozone leads to oxidative stress, which disrupts immune regulation. The thymus gland plays a crucial role in the maturation and differentiation of T lymphocytes, cells essential for the body’s defense and immune tolerance. In the early years of life, the thymus is highly active, but after adolescence, it undergoes a process known as thymic involution. This process involves a reduction in the size and functionality of the thymus, which is gradually replaced by adipose tissue. Ozone pollution exacerbates this involution and impairs the thymus’s proper function. Consequently, thymic cells may alter their function, leading to a decreased production and diversity of T lymphocytes. This decrease contributes to the establishment of chronic inflammatory states, autoimmunity, and a reduced capacity to respond to infections. Immune dysfunction and chronic inflammation can further result in the development and progression of neurodegenerative diseases. Moreover, thymic involution, exacerbated by environmental factors and oxidative stress, negatively impacts overall immunity and accelerates the progression of degenerative diseases over time. This review aims to explore the relationship between oxidative stress and its impact on the thymus gland. We hypothesize that understanding the relationship between ozone pollution and disruption of the redox–immune axis is crucial for understanding the role of the thymus in senescence and neurodegenerative diseases. To explore this topic, we conducted a review from 2006 to 2025, utilizing several databases, including PubMed, Scopus, Google Scholar, EBSCO, and Web of Science.

## 1. Introduction

Ozone pollution is a significant public health concern due to its association with various degenerative diseases. It has been widely demonstrated that repeated exposure to low doses of ozone, such as that occurring on days or periods of high ozone pollution, leads to an increase in emergency department visits for chronic conditions [1]. Ozone is a secondary pollutant formed by the effect of ultraviolet light on other gases, such as nitrogen oxides or volatile organic compounds, present in the troposphere due to human activities and the burning of fossil fuels [2]. When inhaled, ozone produces reactive oxygen species (ROS) that reach the lungs and brain, as well as cytokines that are distributed throughout the body via the blood [3,4]. Ozone pollution is a significant public health concern due to its association with various degenerative diseases. Research has shown that repeated exposure to low doses of ozone, such as that experienced during days or periods of high ozone levels, can lead to an increase in emergency department visits for chronic health conditions. According to surveys by the World Health Organization, exposure to environmental ozone pollution is responsible for approximately 7 million deaths annually [5].

Repeated inhalation of ozone leads to a chronic state of oxidative stress, which is linked to chronic degenerative diseases and plays a significant role in their progression and development [6]. Studies indicate that the redox state influences the immune system’s response; thus, an imbalance in oxidation–reduction can disrupt the regulation of the inflammatory response, a critical defense mechanism in the resolution of the disease [7]. This inflammatory response, which is typically self-limiting and reparative, can lose its self-limiting capacity, becoming persistent over time. Lymphatic organs play an important role in an organism’s immune system. Some of these organs, such as the thymus, spleen, and spinal cord, help maintain immune homeostasis. The thymus is essential in this immune response, both for ensuring an adequate response through thymocytes and for regulating its own cells under chronic oxidative stress [8].

The thymus gland is a crucial organ in the development and maturation of the immune system. Research indicates that it is particularly susceptible to oxidative damage, which can result in several issues, including thymic atrophy [9], thymocyte apoptosis [10], and impaired T lymphocyte development [11]. Additionally, oxidative stress can alter the stromal microenvironment [12], disrupt positive and negative selection processes [13], and contribute to early thymic involution [14]. The aim of this review is to explore the relationship between oxidative stress and its impact on the thymus gland. Understanding the relationship between ozone pollution and disruption of the redox–immune axis is crucial for understanding the role of the thymus in senescence and inflammatory processes involved in neurodegenerative diseases.

## 2. Thymus Physiology

**Figure 1:**
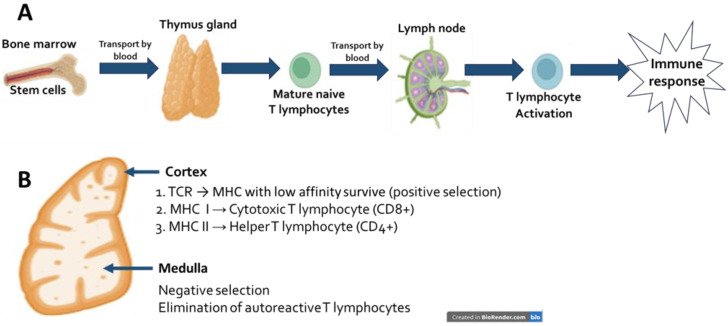
Physiology of the thymus. (**A**) Lymphocytes: maturation and activation. In the bone marrow, common lymphoid progenitors migrate to the thymus, where they differentiate into mature but naïve helper T lymphocytes (CD4^+^) or cytotoxic T lymphocytes (CD8^+^). These cells then migrate to secondary lymphoid organs, such as lymph nodes, where they become activated and participate in the immune response. (**B**) Cortex. 1. T cell receptor (TCR): T lymphocytes that recognize the self-MHC complex with low affinity are the ones that survive (positive selection). 2. CD4^+^ T lymphocytes recognize antigens presented by MHC class II molecules on antigen-presenting cells such as macrophages, dendritic cells, and B lymphocytes. 3. CD8^+^ T lymphocytes recognize antigens presented by MHC class I molecules on all nucleated cells. Medulla. Lymphocytes that recognize self-antigens with high affinity are eliminated (negative selection), thus preventing autoimmunity (Created in BioRender.com. M. Valdes-Fuentes, 2025. https://app.biorender.com/illustrations/60d5139f2a12ca00a68e472d?slideId=8563537e-9ecc-4632-9afc-00afd7e3699c.

Located in the anterior mediastinum, the thymus is most active during childhood. However, beginning at puberty, it undergoes a process called involution, which is characterized by a decrease in size and a gradual replacement of its tissue with adipose tissue. The thymus gland is a primary lymphoid organ that, in addition to participating in lymphocyte maturation, plays a crucial role in the adaptive immune response. It is composed of an outer cortex, where immature thymocytes are located, and an inner medulla that contains mature thymocytes, along with a set of thymic epithelial cells called cortical or medullary cells. Other cell types, such as macrophages and dendritic cells, are also found [15,16]. The thymus is located in the anterior mediastinum and exhibits maximum activity during childhood; however, from puberty onwards, it undergoes a process of involution, characterized by a reduction in size and progressive replacement by adipose tissue [17,18]. During thymus involution, its function declines, leading to reduced production of virgin T lymphocytes, decreased organ structure, increased susceptibility to infections, and the onset of autoimmune diseases with aging. This process ultimately contributes to immunosenescence [19]. However, research indicates that thymic remnants may still play a role in regulating certain aspects of immunological homeostasis. For instance, Kooshesh and collaborators found that patients who had their thymus removed experienced a higher incidence of autoimmune disorders [20]. The thymus plays a crucial role in the immune system by maturing thymocytes that migrate from the bone marrow (see Figure 1). This maturation process is essential for recognizing foreign pathogens, thereby maintaining the organism’s homeostasis in the face of disease [21] and preventing the onset of autoimmune disorders [20]. This organ plays a crucial role in the immunity of organisms by maturing thymocytes that migrate from the bone marrow (Figure 1). This maturation process ensures the recognition of foreign pathogens and, therefore, the organism’s homeostasis in the face of a pathological process [21]. Another function of the thymus is positive and negative selection, which are fundamental processes that allow for the recognition of self-specific major histocompatibility complex molecules, as well as the elimination of autoreactive T cells that can promote the development of autoimmune diseases [22]. All of these functions are crucial for maintaining the proper functioning of the immune system.

During the aging process, the thymus gradually develops a pro-oxidant environment as antioxidant enzymes decline, leading to an increase in ROS. Thymic epithelial cells (TECs) undergo senescence and release molecules that negatively affect the cellular and tissue microenvironment. This phenomenon is associated with the senescence-associated secretory phenotype (SASP), which is characterized by the secretion of cytokines such as interleukin-6 (IL-6), Tumor Necrosis Factor-α (TNF-α), and interleukin-1β (IL-1β) [23]. The rise in cytokine levels is further accompanied by the loss of the transcription factor known as autoimmune regulator (AIRE), a transcription factor found in the medulla and thymus. Additionally, oxidative stress leads to a decrease in forkhead box N1 (FOXN1) levels, resulting in impaired central tolerance [24]. Together, these changes create an inflammatory microenvironment that obstructs the generation of stable regulatory T cells (Tregs).

**Figure 2:**
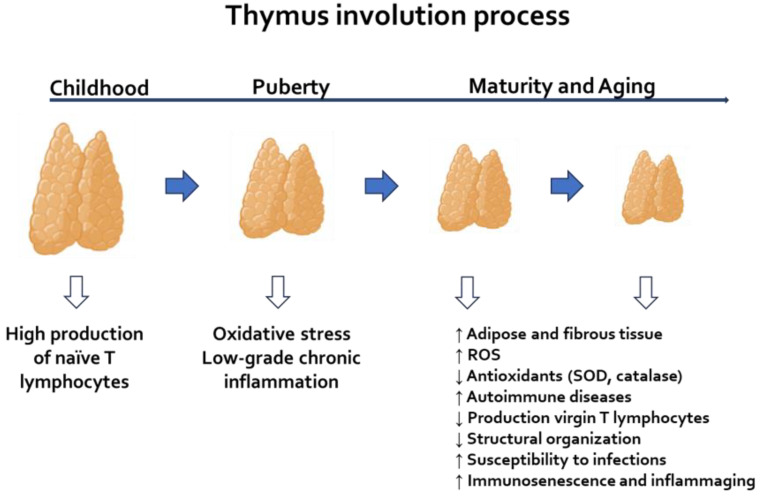
Thymus involution process. During childhood, the thymus is large, and its cortex is rich in naïve T lymphocytes. The process of involution begins at puberty, progresses gradually, and triggers several responses, including increased oxidative stress and low-grade inflammation, ultimately leading to a state characterized by immunosenescence and inflammaging.

The oxidizing microenvironment has significant immunological consequences, including accelerated thymus involution (Figure 2) and the loss of naive thymocytes. This environment also alters thymic Tregs (tTregs) [25], primarily due to the instability of Foxp3 in conditions of increased ROS. Such changes contribute to systemic inflammation and the progression of degenerative diseases, including atherosclerosis, neurodegeneration, and type 2 diabetes [6]. To counter these effects, thymic antioxidant defenses—such as SOD, CAT, GPx, peroxiredoxins (Prx), GSH, NRF2, FOXO, and SIRT1/3—play a vital role in maintaining redox balance [8,26]. This balance is essential for T cell maturation and the immunological tolerance necessary for their proper function. As people age, various functions decline due to the development of an inflammatory microenvironment, which worsens the process of “inflammaging” and the overall deterioration of the immune system. This age-related thymic inflammaging leads to a decrease in epithelial tissue and increase thymic fat, resulting in a loss of T cell diversity. Consequently, the immune system’s ability to recognize new antigens diminishes. This process is further complicated by the persistent release of proinflammatory cytokines, creating a vicious cycle where oxidative stress fuels thymic inflammation. This inflammation accelerates thymic dysfunction and contributes to ongoing systemic low-grade inflammation, which then restarts the cycle [23,27]. Thymic involution has been associated with neurodegenerative disorders, including Alzheimer’s disease and Parkinson’s disease, as well as autoimmune diseases like multiple sclerosis. These conditions involve inflammation and a decline in immune function. Changes in immune function indicate a direct impact on thymus physiology and its aging process [28]. Aging tends to maintain the body in a state of chronic subclinical inflammation, which contributes to a higher incidence and progression of various age-related pathologies, including neurodegenerative diseases. Metabolic disorders, such as obesity and metabolic syndrome, are closely linked to the thymus, as hormones like leptin and ghrelin can influence thymic homeostasis [29]. Moreover, both malnutrition and obesity can induce oxidative stress, leading to changes in the thymus [30].

## 3. The Role of the Thymus in Immune Function, Redox Activity, and Immune Tolerance

### 3.1. T Lymphocytes Selection in the Thymus

One of the functions of the thymus is to mature T lymphocytes that originate in the bone marrow. One of the key processes that occurs in the thymus is positive selection, which enables thymocytes to recognize self-major histocompatibility complex (MHC) molecules [31].

In contrast, negative selection takes place in the thymus, where autoreactive thymocytes are eliminated through apoptosis because they recognize self-peptides presented by TECs and dendritic cells with high affinity [28]. This process, known as central tolerance, relies on the expression of specific autoantigens in the thymus. The AIRE plays a crucial role in this process by regulating the presentation of various peptides derived from peripheral organs to thymocytes. This thereby contributes to the elimination of autoreactive thymocytes and prevents the development of autoimmunity [32].

### 3.2. Oxidative Stress and Antioxidant Enzymes in Thymus Function

The thymus has a unique redox microenvironment. Some studies indicate that TECs exhibit low catalase activity [33]. Elevated hydrogen peroxide levels can lead to irreversible thymic atrophy, a condition that is accelerated by stromal catalase deficiency [34]. Additionally, the thymus has various mechanisms to maintain redox balance and mitigate the effects of free radicals (see Table 1 and Table 2). Thymocytes are particularly sensitive to oxidative stress, as they generate ROS during maturation [8]. TECs also rely on a proper redox balance for both positive and negative selection. Moreover, a decline in both enzymatic and non-enzymatic antioxidant defenses with age accelerates thymic involution and contributes to the development of inflammation (Table 3) [27,35,36].

Transcriptional regulation of antioxidant defenses is mediated by nuclear factor erythroid 2 (NFE2L2), commonly known as Nrf2. This transcription factor activates the expression of genes responsible for producing superoxide dismutase (SOD), catalase (CAT), glutathione peroxidase (GPx), heme oxygenase-1 (HO-1), and NAD(P)H quinone dehydrogenase 1 (NQO1) [8,26]. Additionally, forkhead box proteins (FOXO), including FOXO1, play a crucial role in cellular metabolism and resistance to oxidative stress by regulating genes related to glutathione (GSH), SOD2, and catalase. These FOXO proteins are inhibited by the protein kinase B/mechanistic Target of Rapamycin (AKT/mTOR) pathway, particularly during the process of inflammaging [23,24,26]. Moreover, sirtuins 1 and 3 (SIRT1 and SIRT3) are part of a family of proteins that are located in both the nucleus and the cytoplasm. They act as physiological modulators of metabolism, contributing to mitochondrial detoxification by regulating SOD2 and CAT, and thereby promoting cellular longevity [26].

There is low catalase enzyme activity in TEC, which is responsible for breaking down hydrogen peroxide. It has been suggested that the intracellular environment of the thymus contains relatively high levels of hydrogen peroxide. Moderate levels of this compound have been found to play a role in signaling functions, such as inducing autophagy in TECs. Research conducted by Semwal and collaborators on the transgenic overexpression of catalase in thymic stromal cells revealed a decrease in autophagy, a delay in the elimination of autoreactive thymocytes, and the infiltration of spontaneous lymphocytes into some peripheral organs, leading to signs of autoimmunity [33]. This suggests that oxidative stress is necessary for proper thymic function; however, excessive oxidative stress can harm the thymic microenvironment.

Griffith and collaborators (2015) examined metabolic damage and premature aging in the thymus. They concluded that, even though there is a decrease in catalase levels, thymic atrophy continues, albeit at a slower rate, regardless of antioxidant levels [34]. Developing thymocytes generate ROS as metabolic byproducts during their proliferation and differentiation. Stromal cells, such as endothelial cells (ECs), have low catalase levels, allowing small amounts of ROS to accumulate. These ROS function as signals or messengers; however, if their levels exceed the regulatory capacity of the surrounding environment, they can lead to deoxyribonucleic acid (DNA) damage and other forms of cellular harm. Some studies indicate that, in ECs, oxidative DNA damage accumulates with age, potentially disrupting organ function [34,53]. Such genetic changes directly impact the structural integrity of the thymus [54,55]. As microenvironmental disorganization occurs, the functional areas of the cortex and medulla diminish with age. Additionally, the thymus is an endocrine organ that produces hormones, including thymosin, thymopoietin, and thymulin, which may have systemic effects on immunity and potentially on metabolism [56,57,58].

## 4. Thymic Involution Caused by Oxidative Stress

Thymic involution with age is a complex and multifactorial process; however, several studies indicate that oxidative stress plays a central role in this phenomenon. As we age, ROS accumulate in tissues due to metabolic imbalances and reduced functionality of antioxidant systems. Research using animal models has shown that an increase in oxidative stress accelerates thymic atrophy, while enhancing antioxidant defenses can mitigate this effect [8]. For example, a study involving exposure to high doses of ozone for several hours significantly accelerated thymic involution, resulting in a noticeable reduction in thymic size [59,60]. Additionally, studies have shown that inducing oxidative damage to mitochondrial DNA in mice results in a decrease in thymus size, accompanied by an increase in apoptosis and cellular senescence. However, this adverse effect was reversed after administering an antioxidant agent [61]. Moreover, research on prematurely aged rats treated with D-galactose revealed thymic atrophy and elevated markers of oxidative stress. The application of an antioxidant compound derived from the plant *Aralia taibaiensis* reduced the loss of thymic mass and other aging markers by activating the FOXO3a and NRF2 transcription pathways [62]. Other antioxidants, such as plastoquinonyl decyl triphenylphosphonium (SkQ1), have also been found to delay age-related thymic involution [63]. These findings suggest that excessive ROS directly damages cells within the thymic microenvironment, increasing the likelihood of cellular senescence or death, which ultimately leads to a decline in thymic function.

The accumulation of hydrogen peroxide leads to direct DNA damage, a consequence of increased ROS in the thymic microenvironment. This process can lead to cell senescence or cell death. Research on stromal, cortical, and medullary cells in the thymus has shown a decrease in the expression levels of the catalase protein compared to lymphoid cells. This makes these thymic cells more susceptible to hydrogen peroxide accumulation and its associated DNA damage [34]. Various studies have demonstrated that antioxidant supplementation can significantly impact thymic inhibition, particularly with vitamin C. For instance, Uchio and collaborators (2015) conducted experiments with knockout mice lacking senescence marker proteins, finding that vitamin C intake suppressed age-related thymic atrophy [64]. Similarly, another study involving transgenic mice engineered to overexpress catalase in mitochondria showed a reduction in thymic atrophy. This supports the hypothesis that ROS, especially in mitochondria, play a crucial role in triggering thymic degeneration [65]. Therefore, understanding the intracellular redox state of thymic cells is essential for grasping the rate of thymic involution.

At the molecular level, chronic oxidative stress in the thymus activates damage and senescence pathways, leading to an increase in ROS. This increase causes protein oxidation, including the oxidation of the p53 protein, which in turn activates cell cycle inhibitors such as p21 [66,67]. The involvement of cell cycle regulatory proteins has helped us understand that the prolonged activation of the cyclin-dependent kinase inhibitor 1A CDKN1a gene (which encodes p21) induces mitochondrial dysfunction and the further production of ROS through signaling pathways involving growth arrest and DNA damage-inducible protein 45 (GADD45), mitogen-activated protein kinase 14 (MAP14), also known as p38 mitogen-activated protein kinase (p38 MAPK), growth-factor-receptor-bound protein 2 (GRB2), transforming growth factor beta receptor 2 (TGFBR2), and transforming growth factor beta (TGF-β). This rise in ROS results in a continuous DNA damage response, underscoring the crucial role of this pathway in the senescent phenotype [67,68].

## 5. The Role of Oxidative Stress in the Thymus and Its Relationship with Neurodegenerative Diseases

The relationship between chronic oxidative stress in the thymus and neurodegenerative diseases is complex. It involves an increase in reactive species that leads to thymic involution and the subsequent onset of immunosenescence, which accelerates brain aging. This process results in the production of elevated reactive species and a decline in cognitive function [69].

As the thymus ages, it loses its ability to generate naive lymphocytes [17], resulting in a cell repertoire that predominantly consists of senescent memory and effector cells. This shift favors a chronic proinflammatory state known as “inflammaging”, characterized by low-grade inflammation, which impacts the development of neurodegenerative diseases such as Alzheimer’s and Parkinson’s [70]. Additionally, the involuted thymus is less capable of effectively eliminating autoreactive T lymphocytes during negative selection. This failure can lead to systemic attacks that involve the central nervous system [71]. Research in mouse models exhibiting accelerated thymic involution has shown an increase in circulating autoreactive T lymphocytes infiltrating non-lymphoid organs, accompanied by elevated levels of inflammatory cytokines, such as IL-6 and the tumor necrosis factor alpha (TNF-α) [72]. Thymic inflammaging represents a key factor in inflammatory aging. Through pathways involving Nuclear Factor kappa-light-chain-enhancer of activated B cells (NF-κB), mTOR, the NLR family pyrin domain containing 3 (NLRP3) inflammasome, and the Janus kinase/signal transducer and activator of transcription (JAK/STAT) pathway, a chronic inflammatory environment is maintained. This persistent inflammation accelerates various age-related degenerative diseases by activating different inflammatory pathways, as outlined in Table 4 [23].

Oxidative stress plays a significant role in thymic senescence by reducing T cell diversity and central tolerance, primarily due to decreased levels of AIRE and FOXN1, which in turn amplifies inflammation [8,24,79]. Despite Tregs acting as a “brake” on this inflammation, their metabolic and epigenetic identities are susceptible to high levels of ROS and cytokines, such as IL-6. This vulnerability compromises their ability to regulate inflammation effectively [23]. Consequently, this situation contributes to the progression of age-related degenerative diseases that are activated by thymic inflammaging. Chronic, low-grade inflammation leads to a reduction in TECs, increased adipogenesis, and diminished T cell diversity, while simultaneously enhancing levels of proinflammatory cytokines [27]. Tregs (Foxp3^+^ cells) play a crucial role in maintaining tolerance and originate from two primary sources: tTregs and peripheral Tregs (pTregs). Their identity relies on the presence of Foxp3 (which is stabilized by the demethylation of the conserved noncoding sequence 2 (CNS2) locus), as well as factors such as IL-2, TGF-β, and metabolic processes, including fatty acid oxidation (FAO) and oxidative phosphorylation (OXPHOS) [80,81,82]. Moreover, thymic involution results in fewer niches for thymopoiesis, leading to a loss of naive T cell diversity and an expansion of proinflammatory memory effector T cells. Cytokines involved in pathways such as JAK/STAT (specifically IL-6), mTOR, NF-κB, and NLRP3 can accelerate this involution, creating a vicious cycle between thymic involution and increased proinflammatory cytokines, alongside a loss of T cell diversity [26], as shown in Table 5. The number of tTregs decreases due to limited niches, and TECs become dysfunctional. While pTregs can partially compensate under the influence of TGF-β, retinoids, and indoleamine 2,3-dioxygenase (IDO), their efficacy diminishes in the presence of IL-6 and TNF-α [6,83]. Additionally, alterations in chemokines, such as chemokine (C–C motif) ligand 19 (CCL19), CCL21, and chemokine (C-X-C motif) ligand 12 (CXCL12), along with changes in glycobiology related to thymic selection, affect the homing and tissue residency of Tregs [84]. Another crucial factor is the decline in AIRE, which reduces the generation of tissue antigen-specific tTregs, leading to micro-autoinflammation [25,85].

## 6. Multiple Sclerosis and Neurodegenerative Autoimmunity

Multiple sclerosis (MS) is a neurodegenerative autoimmune disease characterized by significant thymic dysfunction, particularly in patients with the relapsing-remitting variant (RRMS). These patients often experience premature thymic involution, with reports of thymus shrinkage in young individuals with RRMS. One potential cause of this condition is a reduced number of naive T cells and an increased proportion of circulating memory and senescent T cells compared to age-matched healthy individuals [88]. The incomplete clearance of autoreactive clones leads to a breakdown of thymic regulation, resulting in an increase in autoreactive T cells. Research has documented the presence of granulocyte-macrophage colony-stimulating factor GM-CSF-secreting effector T cells [89], DR2a- and DR2b-specific CD4+ T cell clones [90], myelin-specific CD8^+^ CD20^+^ memory T cells [91], and regulatory B cells that produce IL-10 and IL-35 [92]. In this context, an involuted thymus damaged by oxidative stress is unable to efficiently eliminate autoreactive cells, thereby facilitating the development of autoimmune diseases in the nervous system and triggering irreversible pathologies. In MS, regulatory T lymphocytes play a central role in suppressing the responses of T helper cells 1 (Th1) and T helper cells (Th17) effector cells. Therefore, disruptions in self-tolerance homeostasis may serve as the primary cause of immunological attacks, inflammation, and neurodegeneration [93]. Consequently, premature thymic involution, possibly due to oxidative stress, leaves individuals with an immune system that mistakenly attacks their own nervous system.

## 7. Alzheimer’s Disease and Immunity

In Alzheimer’s disease (AD), the role of adaptive immunity is quite complex. Research has shown that in patients with AD, abnormal T lymphocyte populations contribute to neuroinflammation and the release of proinflammatory proteins through glial cells that invade the brain. Various risk factors, including apolipoprotein E, α-secretase, β-secretase, γ-secretase enzymes, Tau protein, and neuroinflammation, affect T cell activation [94]. The presence of autoreactive cells indicates that thymic selection was not fully effective in eliminating them. In certain pathological conditions, effector T cell (Teff) subsets become activated against misfolded self-proteins, leading to a breakdown of immune tolerance and an increase in autoreactive T cells. Some studies have found a clonal expansion of CD8+ T effector cells in certain patients with AD, alongside CD4+ T lymphocytes that react against pathological proteins linked to AD. This includes T helper cell subsets, such as Th1, Th2, and Th17, which are identified in microglial cultures. This research suggests that glial proinflammatory responses are primarily driven by Th1 and Th17 cells, while Th2 cells play a regulatory role [71]. Additionally, interleukins involved in the immune response—such as IL-17, IL-1β, and IL-10—as well as the transcription factor NF-κB, exhibit significant changes in the hippocampus of rats exposed to low concentrations of ozone. This exposure also impacts Th1, Th2, and Th17 responses [4,95]. These findings suggest a loss of regulation in the immune response due to oxidative stress induced by ROS resulting from low-dose ozone exposure.

Ozone pollution contributes to oxidative stress and, along with age-related thymic atrophy, can reduce the expression of certain neuronal autoantigens, such as tau and beta-amyloid proteins, in the thymus. This reduction allows for the survival of potentially harmful immune cell clones. Research indicates that thymocytes in transgenic mice models of AD are not always effectively cleared, leading to the infiltration of autoreactive T cells into the brain, which can contribute to neuroinflammation [96,97]. Moreover, it has been observed that deficiencies in T cells can exacerbate the pathology associated with AD due to the accumulation of amyloid proteins [98]. However, T cell transplantation has been shown to alleviate symptoms of AD and assist B cells in producing anti-Aβ antibodies that neutralize the Aβ toxin and hyperphosphorylated tau [99]. Additionally, T cells can function similarly to macrophages, helping to engulf misfolded proteins such as tau.

These findings suggest that a weakened T cell system, as seen with thymic involution, reduces immunological surveillance and control within organs, particularly the brain. This deficiency allows for the buildup of toxic aggregates, leading to a harmful inflammatory process detrimental to the central nervous system. Furthermore, the phenomenon of immunosenescence and the presence of autoimmunity may play roles in AD, as specific T lymphocytes appear to aid microglia in phagocytosing beta-amyloid protein and stimulating responses to produce anti-amyloid antibodies [100].

## 8. Parkinson’s Disease and Oxidative Stress

Parkinson’s disease (PD) is characterized by the loss of dopaminergic neurons and the accumulation of Lewy bodies, which are protein aggregates composed of alpha-synuclein. Key mechanisms involved in this disease include the activation of microglia and the infiltration of T lymphocytes into brain structures, along with evidence of autoimmune processes targeting neuronal antigens [101]. Some studies have suggested that patients with Parkinson’s disease may have T lymphocytes that are autoreactive against alpha-synuclein [102]. The presence of these autoreactive T cells can infiltrate the nervous system and contribute to neuronal cell death. Consequently, the appearance of these lymphocytes before the onset of apparent symptoms could indicate immune inflammation and predict the development of Parkinson’s disease [102].

In PD, T lymphocytes show significant alterations that coincide with immunosenescence. For instance, thymic deterioration due to atrophy results in a limited repertoire of naive T cells, thereby impairing the ability to recognize new antigens. Kouli and collaborators reported a reduction in CD8 terminally differentiated effector memory CD45RA-positive T cells (TEMRA) T lymphocytes among patients in the early stages of the disease in 2021 [103]. This lack of regulation is crucial, as the thymus involutes, altering the composition and quality of the T-cell response. In this context, the presence of fewer Treg and a higher proportion of pro-inflammatory cells, resulting from Th1 and Th17 responses, can exacerbate neuroinflammation and lead to increased neuronal damage [104,105].

## 9. Other Neurodegenerative Diseases

Research on thymic function in relation to PD and AD is plentiful. Still, T cell infiltrations have also been observed in other conditions, such as amyotrophic lateral sclerosis and prion diseases [106,107,108]. Some studies suggest that individuals with Down syndrome may experience accelerated immune aging due to early thymic involution. Additionally, increased oxidative stress in their traumatic brain injuries could contribute to a higher risk of AD-like dementia [109]. Understanding the connection between chronic inflammation, neuronal death, and immune senescence in aging, especially within the thymus, is essential for studying neurodegeneration. This connection arises from an aging system damaged by oxidative stress, which causes a loss of regulation and leads to further harm. As a result, various strategies have been investigated to reverse thymic involution, enhance thymic function, and regulate inflammatory responses. Examples include anti-amyloid vaccines [110] and the transfer of Tregs [111].

There is a significant interaction between the immune system and the central nervous system that relies on effective communication, which is maintained when thymic homeostasis is intact. However, this communication deteriorates in the presence of neurodegeneration. In summary, thymic dysfunction caused by oxidative stress initiates a series of events that lead to immune senescence, chronic inflammation, and ultimately the progression of neurodegenerative diseases.

## 10. Oxidative Stress and Gene Expression

The thymus is composed of a significant number of cortical and medullary epithelial cells, mesenchymal cells, and macrophages, all of which create an appropriate microenvironment for the selection of T lymphocytes. The biochemical and energetic conditions in this environment are carefully balanced; thus, factors such as ROS concentrations, antioxidant levels, oxygen levels, and metabolites can impact the expression of specific genes. Thymic dysfunction and gene dysregulation contribute to the development of neurodegenerative diseases such as Alzheimer’s or Parkinson’s. Key signaling pathways are regulated by important transcription factors, including Nrf2 and NF-κB, as well as longevity-related pathways like FOXO. All of these components are active in thymic cells and help regulate essential genes for homeostasis and tolerance [112].

The thymus plays a crucial role in maintaining physiological levels of oxidative stress, which in turn supports an adaptive mechanism. Semwal and collaborators (2022) demonstrated that low expression of catalase in the thymus plays a role in regulating the central tolerance of T cells [33]. This regulation leads to increased levels of constitutive autophagy, which is crucial for presenting autoantigens and selecting T cells. Consequently, ROS signaling can induce the expression of autophagy-related genes, such as beclin-1 (BECN1) and microtubule-associated proteins 1A/1B light chain 3B (LC3) [113,114,115]. This suggests a connection between oxidative stress and the continuous activation of autophagy genes.

The transcription factor AIRE plays a vital role in eliminating autoreactive immune cells. Mutations in AIRE can lead to autoimmune syndromes characterized by a range of symptoms [116]. This factor is also linked to the aging processes in the thymus, as it promotes the expression of tissue-restricted antigens (TRAs). Additionally, certain antioxidants, such as vitamin D, help regulate the expression of AIRE and TRAs in medullary TECs, thereby actively contributing to the process of thymic aging [117].

During the aging process, the expression of AIRE and various TRAs in medullary TECs significantly decreases. A 2022 study by Hester and collaborators linked this decline in expression to defects in the elimination of autoreactive lymphocytes in aged mice. The reduction in AIRE levels may be caused by multiple factors, including epigenetic changes or a loss of mature TECs, with chronic oxidative stress potentially contributing to this process. Excessive ROS can disrupt the transcription factors or signaling pathways necessary for AIRE expression. For instance, AIRE relies on receptor activator of nuclear factor kappa B ligand (RANKL) and CD40 signaling, and also plays a critical role in maintaining chromatin integrity. Oxidative stress can lead to DNA damage or epigenetic chromatin modifications, resulting in the silencing of specific genes [118,119]. Interestingly, some antioxidants, such as curcumin, have been shown in mouse models to enhance AIRE expression in the thymus, partially reversing the loss of autoantigen transcription. Some antioxidants, such as curcumin, can reduce oxidative stress, which seems to activate the AIRE transcriptional machinery, subsequently reestablishing central tolerance. A 2021 study by Ding and collaborators demonstrated that curcumin rejuvenates thymic involution by increasing the levels of AIRE and promoting the proliferation of TECs. This proliferation suggests that antioxidants may exert a redox-modulating effect, influencing the expression of specific genes crucial for the development of the thymic microenvironment [52]. Redox-sensitive transcription factors play a vital role in these processes. The transcription factor Nrf2 is a key endogenous regulator of antioxidants. ROS activates it and translocates to the nucleus, where it binds to an antioxidant response element (ARE) [120]. In the aging thymus, Nrf2 activation can induce the expression of genes such as CAT, GPx, and HO-1, among others. This activation in thymic cells helps maintain homeostasis in response to stress. Nrf2 activation interacts with NF-κB, leading to a reduction in the expression of pro-inflammatory genes. Therefore, a thymic microenvironment with overexpressed Nrf2 would demonstrate lower levels of local inflammation and an enhanced antioxidant capacity, helping to preserve the expression of essential functional genes. However, it has been observed that oxidative stress can impair the function of Nrf2 through damage or mutations, which often leaves NF-κB active in such situations [112]. In thymic cells, the FOXO3a pathway promotes the expression of genes related to antioxidants and cellular longevity. In models of thymic oxidative stress, activation of FOXO3a is associated with protection against atrophy. Since FOXO3a can induce the expression of specific antioxidant enzymes, such as catalase and SOD2, it indicates that in a low-oxidative thymic microenvironment, FOXO3a remains inactive in a phosphorylated state within the cytosol. However, under moderate stress, FOXO3a can be translocated to the nucleus, where it regulates the expression of these protective genes. Additionally, FOXO3a/Nrf2 signaling has been documented, suggesting a protective role in delaying the aging process.

## 11. Conclusions

Future research should focus on elucidating the molecular mechanisms by which chronic exposure to certain pollutants, especially ozone, induces systemic oxidative stress and alters thymic homeostasis. It would be particularly interesting to study how ozone affects the architecture and function of the thymus, as well as the differentiation and immunological competence of T cells. Research has shown that repeated exposure to low doses of ozone can lead to a chronic state of oxidative stress. The ROS increase disrupts the regulation of the immune response, which in turn affects the inflammatory response. As a result, the thymus gland loses its ability to manage inflammation effectively. Over time, thymocytes—especially Tregs cells—become exhausted. The alteration of the thymus’ physiological functions by T cell exhaustion enables degenerative processes to advance, a phenomenon commonly associated with aging. Consequently, this shift affects the regulation of inflammatory processes in various organs and systems, contributing to the development and progression of autoimmune and neurodegenerative diseases.

## Figures and Tables

**Table 1:** Temporal development of thymus function depending on redox state.

Key Process	Redox State	Consequence	References
Positive Selection Self-	Redox Balance	MHC Recognition	[31]
Negative Selection (AIRE)	Redox Balance	Elimination of Autoreactive Cells	[32]
Thymus in the stage of youth homeostasis	Redox Balance Moderate ROS	Signaling and Functional Autophagy	[8]
Chronic Oxidative Stress	Elevated ROS	DNA Damage, Loss of Immune Tolerance	[37]
Atrophy and Dysfunction	Loss of Redox Balance Decreased Catalase	Structural Disorganization	[38,39]
Endocrine Involution	Chronic Oxidative Stress State Persistent ROS	Immunosenescence and Autoimmunity Inflammation	[27,35,36]

**Table 2:** Enzymatic antioxidant defenses of the thymus.

Enzyme	Main Location in the Thymus	Key Function	Elevance in Aging	References
Superoxide dismutases (SOD1, SOD2, SOD3)	SOD1 (cytoplasm), SOD2 (mitochondria), SOD3 (extracellular space)	Convert O_2_^−^ → H_2_O_2_	↓ Activity with age, more mitochondrial damage	[30]
Catalase (CAT)	Peroxisomes, TECs, and thymocytes	Degrades H_2_O_2_ → H_2_O + O_2_	Declines with age, promotes SASP and TEC senescence	[40]
Glutathione peroxidases (GPx1-4)	Cytoplasm and mitochondria	Scavenge H_2_O_2_ and lipid peroxides using GSH	With thymic involution, ↓ GSH and GPx activity	[41]
Peroxiredoxins (Prx I–VI)	Cytoplasm, nucleus, and mitochondria	Reduction of H_2_O_2_ to physiological levels, buffering redox signals	Redox alteration leads to chronic stress	[42]
Thioredoxin reductase (TrxR)	Cytoplasm/mitochondria	Maintains proteins in a reduced state, supports Prx	Its alteration induces thymic apoptosis.	[43,44]

Downward arrows indicate a decrease.

**Table 3:** Non-enzymatic antioxidant defenses of the thymus.

Molecule	Function	Changes with Age	References
Glutathione (GSH)	Main intracellular redox buffer, maintains reduced protein status.	Decreases with age, promotes Treg dysfunction	[45]
Antioxidant vitamins (C, E, A)	Neutralize ROS and protect cell membranes	Decreased bioavailability with aging.	[46,47,48]
Uric acid, bilirubin	Secondary endogenous antioxidants	May be altered in chronic inflammatory states	[49,50]
Dietary polyphenols (experimental)	Enhance NRF2 and antioxidant enzymes.	Supplementation in animal models improves thymic function	[51,52]

**Table 4:** Signaling pathways in thymic inflammation.

Pathway	Stimulants/Activators	Effects on the Thymus	Systemic Consequences	References
NF-κB	TNF-α, IL-1β, Toll-like Receptor (TLRs), ROS	↑ Proinflammatory cytokine expression, TEC senescence, SASP	Chronic inflammation, perpetuation of thymic involution	[73]
mTOR	Nutrients, IL-7, Insulin-like Growth Factor 1 (IGF-1), ROS	Inhibits autophagy, promotes thymic lipogenesis/adipogenesis, ↑ proinflammatory effector T cells	Decreases thymic regeneration, accelerated aging, reduced T cell repertoire.	[74]
NLRP3 Inflammasome	ROS, Damage-Associated Molecular Patterns (DAMPs), mitochondrial damage	Processes IL-1β, IL-18, and TECs and thymic macrophages	Amplifies systemic inflammation, contributes to osteoarthritis, neurodegeneration	[75]
JAK/STAT (IL-6/STAT3)	IL-6, IL-10, interferons	Persistent proinflammatory signaling in thymocytes and stroma	Insulin resistance, metabolic dysfunction, vascular inflammation	[76]
IL-6/C-reactive Protein (CRP)/Hepcidin axis	Inflammaging-induced hepatic IL-6.	↓ Iron homeostasis, ↑ anemia of inflammation	Chronic immune and metabolic impairment	[77]
Cyclic GMP-AMP synthase-stimulator of interferon genes (cGAS–STING) (emerging)	Cytosolic DNA due to nuclear/mitochondrial damage	Induction of interferon (IFN) type I, chronic antiviral/inflammatory state	Sterile immune activation, increased cellular senescence	[78]

Upward arrows indicate an increase, and downward arrows indicate a decrease.

**Table 5:** Oxidative stress, thymic inflammation and Treg cells.

Axis	Key Mechanisms	Signaling Pathways	Consequences	Impact on Tregs	References
**Oxidative stress** **(OS)**	↑ ROS/RNS, mitochondrial and DNA damage, antioxidant depletion	NF-κB, p38/JNK, cGAS-STING, NLRP3, ↓ NRF2/FOXO, ↓ mitophagy	SASP, stromal senescence, chronic inflammation	ROS/HIF-1α destabilizes Foxp3, ↑ glycolysis, ↓ FAO/OXPHOS, loss of suppressor function	[86]
**Thymus and inflammaging**	Thymic involution (↓ TECs, ↑ adipogenesis, ↓ AIRE), loss of T diversity	NF-κB, mTORC1, JAK/STAT (IL-6), NLRP3	Reduced T repertoire, skewed clonality, microautoimmunity	↓ tTregs (by fewer niches), pTregs compensate but are less effective under IL-6/TNF-α	
**Chronic inflammation**	Persistent cytokines: IL-6, IL-1β, TNF-α, CRP	JAK/STAT3, NF-κB, inflammasome NLRP3	Inflammatory amplification, microglia activation, sarcopenia, atherosclerosis	Inflammatory environment erodes epigenetic stability of Foxp3, favoring Treg leads to Th17 transition	
**Treg metabolism**	Prefer FAO/OXPHOS and low glycolysis	AMPK, SIRT1/3, mTORC1 (dose-dependent), STAT5	Functional stability and metabolism-dependent suppressor	↑ ROS and hyperactive mTOR leads to Treg’s loss of identity ; Antioxidants and FAO leads to Foxp3 preservation	[87]
**Biomarkers**	GSH/GSSG, 8-oxo-dG, protein carbonyls	—	Indicators of OS and aging	Foxp3, pSTAT5, CNS2 methylation, mitochondrial ROS	
**Potential interventions (research)**	Thymic rejuvenation, NRF2 activators, mTOR control, AMPK activators, NLRP3 inhibitors	—	Slow inflammation and improve immune homeostasis	Low-dose IL-2, FAO/OXPHOS support, maintain demethylated Foxp3 locus	

Upward arrows indicate an increase, and downward arrows indicate a decrease.

## Data Availability

The original contributions presented in this study are included in the article. Further inquiries can be directed to the corresponding author.

## Abbreviations

The following abbreviations are used in this manuscript:

8-oxo-dG	8-Oxo-2′-deoxyguanosine
AD	Alzheimer’s disease
AIRE	Autoimmune regulator
AKT	Protein kinase B
BECN1	Beclin-1
CAT	Catalase
CCL	Chemokine (C–C motif) ligand
CD4	Helper T cells
CD8	Cytotoxic T-lymphocyte
CDKN1A	Cyclin-Dependent Kinase Inhibitor 1A
cGAS–STING	Cyclic GMP-AMP synthase- stimulator of interferon genes
CNS2	Conserved Noncoding Sequence 2
CRP	C-reactive Protein
CXCL12	Chemokine (C-X-C motif) ligand 12
DAMPs	Damage-Associated Molecular Patterns
DNA	Deoxyribonucleic acid
DR2	Human Leukocyte Antigen-DR2
ECs	Endothelial cells
FAO	Fatty acid oxidation
FOXN1	Forkhead box N1
FOXO	Forkhead box proteins
GADD45	Growth Arrest and DNA Damage-inducible protein 45
GM-CSF	Granulocyte-Macrophage Colony-Stimulating Factor
GPx	Glutathione peroxidase
GRB2	Growth-factor-receptor-bound protein 2
GSH	Glutathione
GSSG	Glutathione disulfide
HO-1	Heme oxygenase-1
IDO	Indoleamine 2,3-dioxygenase
IFN	Interferon
IGF-1	Insulin-like Growth Factor 1
IL	Interleukin
JAK/STAT	Janus Kinase/signal transducer and activator of transcription
LC3	Microtubule-associated proteins 1A/1B light chain 3B
MAP14	Mitogen-activated protein kinase 14
MHC	Major histocompatibility complex
MS	Multiple sclerosis
mTOR	Mechanistic Target of Rapamycin
NF-κB	Nuclear Factor kappa-light-chain-enhancer of activated B cells
NLRP3	NLR family pyrin domain containing 3
NQO1	NAD(P)H quinone dehydrogenase 1
Nrf2	Nuclear factor erythroid-derived 2
OS	Oxidative stress
OXPHOS	Oxidative phosphorylation
p38 MAPK	p38 mitogen-activated protein kinase
PD	Parkinson’s disease
Prx	Peroxiredoxins
pTregs	Peripheral Tregs
RANKL	Receptor activator of nuclear factor kappa B ligand
RNS	Reactive nitrogen species
ROS	Reactive Oxygen Species
RRMS	Relapsing-remitting variant
SASP	Senescence-associated secretory phenotype
SIRT	Sirtuins
SkQ1	Plastoquinonyl decyl triphenylphosphonium
SOD	Superoxide dismutase
TECs	Thymic epithelial cells
Teff	Effector T cell
TEMRA	Terminally differentiated effector memory CD45RA-positive T cells
TGFBR2	Transforming Growth Factor Beta Receptor 2
TGF-β	Transforming Growth Factor beta
Th	T helper cells
TLRs	Toll-like Receptor
TNF-α	Tumor Necrosis Factor alpha
TRAs	Tissue-restricted antigens
Tregs	Regulatory T cells
tTregs	Thymic Tregs
